# Heterozygous loss of *SRRM1* may be associated with neurodevelopmental phenotypes and anomalies in cell growth and neurite morphology

**DOI:** 10.1038/s41431-025-01966-y

**Published:** 2025-10-27

**Authors:** Melek Firat Altay, Anne Gregor, Dominique Braun, Claudine Rieubland, Matthias Gautschi, Eveline Perret Hoigné, Rike Schiller, Boris Keren, Alejandra Afenjar, Undiagnosed Diseases Network, Julian A. Martinez-Agosto, Jill A. Rosenfeld, Julian A. Martinez-Agosto, Jill A. Rosenfeld, Christiane Zweier

**Affiliations:** 1 Department of Human Genetics, Inselspital, University of Bern, Bern, Switzerland; 2 Department for Biomedical Research (DBMR), University of Bern, Bern, Switzerland; 3 Division of Paediatric Endocrinology, Diabetology and Metabolism, Department of Paediatrics, and Institute of Clinical Chemistry, Inselspital, Bern University Hospital, University of Bern, Bern, Switzerland; 4 Division of Neuropediatrics, Department of Paediatrics, Inselspital, Bern University Hospital, Bern, Switzerland; 5 Department of Diagnostic, Interventional and Paediatric Radiology, University of Bern, Inselspital Universitätsspital Bern, Bern, Switzerland; 6 APHP, Sorbonne Université, Département de génétique médicale, GH Pitié Salpêtrière, Paris, France; 7 Clinical Genetics Unit, Reference Center for Cerebellar Malformations and Congenital Diseases, Armand-Trousseau Hospital, APHP, Sorbonne University, Paris, France; 8 Department of Human Genetics, UCLA, Los Angeles, CA USA; 9 Department of Molecular and Human Genetics, Baylor College of Medicine, Houston, TX USA; 10 Baylor Genetics Laboratories, Houston, TX USA

**Keywords:** Neurodevelopmental disorders, Genetics research

## Abstract

Serine/arginine repetitive matrix protein 1 (SRRM1) is a key component of spliceosomes and plays various roles in messenger RNA processing. To date, its function in the nervous system has not been elucidated, and germline variants in *SRRM1* have not yet been implicated in disease. Through international collaboration, we have identified three individuals harbouring heterozygous truncating variants in *SRRM1*, presenting variably with developmental delay, intellectual disability, short stature, behavioural and skeletal anomalies, and facial dysmorphism. Two of the variants occurred de novo, while the third could not be tested in the parents. Reduction of SRRM1 to 50% in SKNBE2 cells by introducing a truncating variant via CRISPR-Cas9 editing, followed by differentiation into neuron-like cells, resulted in impaired cell proliferation, migration, and neurite outgrowth compared to wild-type cells. Additionally, the role of *SRRM1* in nervous system development and functioning was investigated in vivo using a *Drosophila* model. Pan-neuronal knockdown of the orthologue *Srrm1* led to reduced viability, while motoneuronal knockdown impaired gross neurological function. Taken together, we provide multiple lines of evidence that loss of *SRRM1* is associated with nervous system-related phenotypes, and that its haploinsufficiency may be causative for a neurodevelopmental disorder.

## Introduction

The serine/arginine repetitive matrix 1 (SRRM1) protein, also known as serine/arginine repeat-related nuclear matrix protein of 160 kilodaltons (SRm160), is encoded by the *SRRM1* gene and is a nuclear matrix-associated splicing factor that constitutes a component of the spliceosome [1]. Spliceosomes are complex and dynamic molecular machines responsible for RNA splicing [2], during which introns are removed from the precursor messenger RNA (pre-mRNA), and exons are joined together to form mature messenger RNA (mRNA). The major spliceosome, responsible for the bulk of splicing events, is assembled from uridine-rich small nuclear ribonucleoproteins (snRNPs), along with numerous non-snRNP proteins, including serine-arginine-rich (SR) proteins such as SRRM1 and other splicing factors [3].

SRRM1 spans 904 amino acids and features a proline-tryptophan-isoleucine (PWI) domain that facilitates binding to nucleic acids [4]. It primarily localises to nuclear speckles, where it forms a complex with serine/arginine repetitive matrix 2 (SRRM2) [5], SR proteins [5, 6], and interacts with snRNPs [7], acting as a mediator in spliceosome stabilisation. It plays a pivotal role in various stages of mRNA metabolism [8], including constitutive and alternative splicing [7], 3’-end cleavage and polyadenylation [4, 9], formation of exon-exon junction complexes (EJC) [10], mRNA cytoplasmic export, and the initiation of nonsense-mediated mRNA decay (NMD) [11].

A role for *SRRM1* has so far been reported in liver [12] and prostate [13] cancers. Its overexpression is linked to aberrant splicing of *CD44* and increased malignancy [14]. However, germline variants in *SRRM1* have not yet been implicated in any disease. Furthermore, despite its expression in the brain, according to the Genotype-Tissue Expression portal (www.gtexportal.org), a role in neurodevelopment and nervous system function or dysfunction has not yet been characterised. Here, we report three individuals with variable neurodevelopmental and growth phenotypes who carry heterozygous truncating variants in *SRRM1*, two of which occurred de novo. Altered cell growth and neurite development in *SRRM1*-deficient neuron-like cells, along with impaired gross neurological functioning in *Drosophila melanogaster* following motoneuronal knockdown (KD) of *Srrm1*, support *SRRM1* as a candidate gene for a neurodevelopmental disorder (NDD).

## Materials and methods

### Molecular and clinical data

Cases with *SRRM1* truncating variants were identified via GeneMatcher (https://genematcher.org/) [15] and personal communication. (Trio) exome sequencing was conducted according to standard diagnostic or research procedures at the respective centres, with research procedures approved by the relevant institutional review boards. Informed written consent for genetic testing and publication of molecular and clinical data was obtained from the parents or legal guardians of the affected individuals. A study for the characterisation of rare diseases was approved by the Cantonal Ethical Review Board Bern. Identified variants were submitted to ClinVar.

### Cell culture

SKNBE2 cells (ATCC, Gaithersburg, Maryland, US) were maintained under sterile conditions at 37 °C and 5% CO_2_ with appropriate humidity. Cells were cultured in Dulbecco’s Modified Eagle Medium/Nutrient Mixture F-12 (DMEM/F-12) supplemented with 10% foetal bovine serum (FBS) and 1% penicillin-streptomycin, and passaged every 3–4 days. Differentiation was induced in low-serum medium (1% FBS) with 10 µM retinoic acid and 25 µM caffeic acid over 10 days, as described elsewhere [16].

### CRISPR-Cas9 editing

*Plasmid construction:* Single guide RNAs (sgRNAs) targeting *SRRM1* were designed using sequences from the UCSC Genome Browser (GRCh38/hg38, December 2013) (https://genome.ucsc.edu/). An sgRNA targeting exon 9 (GCCCCCCTCGGAAAACTCGT), an exon consistently present in all *SRRM1* transcript isoforms, was generated using the CRISPOR web tool (http://crispor.tefor.net/) and cloned into a pX330-based vector (AddGene, Watertown, MA, US), which contains an additional eGFP ORF. The construct was validated using the T7 endonuclease 1 (T7E1) mismatch detection assay [17]. *Transfection and sorting:* Metafectene Pro (Biontex, Munich, Germany), a lipid-based transfection reagent, was used to deliver the construct into SKNBE2 cells. Single eGFP-positive cells, indicating successful transfection, were isolated by fluorescence-activated cell sorting (FACS) on a MoFlo Astrios cell sorter (Beckman Coulter, Brea, CA, US) 48 h after transfection. *Validation:* Expanded colonies were harvested, and genomic DNA was extracted for Sanger sequencing to confirm variants. Results were analysed using the TIDE web tool (http://shinyapps.datacurators.nl/tide/), and positive clones were selected. Three *SRRM1* compound heterozygous (cHet) lines (cHet 1–3) were established, each carrying the heterozygous truncating variant p.(Arg386Serfs*2) and an in-frame variant on the second allele - either p.(Arg386_Ser389del) or p.(Pro382_Arg387del) (NM_005839.4) (Supplementary Table S1). These cHet lines were matched with three wild-type (WT) lines processed through the same CRISPR workflow and used as controls.

### Western blotting

Cells were washed in Dulbecco’s phosphate-buffered saline (DPBS), extracted in Tris-buffered saline (50 mM Tris, 150 mM NaCl, pH7.5) with 2% sodium dodecyl sulphate, 1:100 Halt protease inhibitor cocktail (TF #78429) and 1 mM phenylmethylsulfonyl fluoride (AppliChem, Darmstadt, Germany), then boiled at 90 °C for 20 min. Samples containing 10 µg of protein, measured using the Pierce bicinchoninic acid assay kit (ThermoFisher, Waltham, MA, US), were run on 4–20% Mini-PROTEAN Tris-glycine gels (Bio-Rad, Hercules, CA, US) and transferred to a 0.22 μm nitrocellulose membrane using a semi-dry transfer system (Bio-Rad). Membranes were incubated with anti-SRRM1 rabbit polyclonal (1:2000; Abcam #ab221061, Cambridge, UK) or anti-alpha-tubulin DM1A mouse monoclonal (1:10,000; Abcam #ab7291) primary antibodies, followed by HRP-conjugated goat anti-rabbit (1:15,000; Bio-Rad) or goat anti-mouse (1:15,000; Abcam) secondary antibodies. Experiments were repeated at least three times. Image analysis was performed on Image Lab (Bio-Rad) where densitometric values for SRRM1 bands were normalised to corresponding alpha-tubulin bands. A one-sample *t*-test (hypothetical value = 100) was applied for statistical analysis.

### Immunocytochemistry (ICC)

SKNBE2 cells grown on poly-D-lysine (PDL)-coated coverslips were washed in DPBS, fixed in 4% paraformaldehyde (PFA) for 20 min at room temperature (RT), and permeabilised/ blocked in PBS with 3% bovine serum albumin and 0.1% Triton for 1 h at RT. Cells were incubated with primary antibodies - anti-SRRM1 rabbit polyclonal (1:100; Abcam #ab221061), anti-alpha-tubulin DM1A mouse monoclonal (1:800; Abcam #ab7291), or anti-SC35 (1:200; Abcam #ab11826) - diluted in blocking solution for 2 h at RT. After three washes, secondary antibodies - goat anti-mouse Alexa Fluor 488 (1:2000; ThermoFisher) and donkey anti-rabbit Alexa Fluor 546 (1:500; ThermoFisher) – were applied for 1 h at RT. Nuclei were stained with DAPI (1:50,000; ThermoFisher). Coverslips were washed and mounted with AquaPolyMount (Polysciences, Warrington, PA, US). Images were captured using the Axio Imager.Z2 microscope (Zeiss, Oberkochen, Germany) and analysed with Zen Digital Imaging software (Zeiss).

### Cell growth assay

Growth of non-differentiated and differentiated SKNBE2 cells was assessed using the CyQUANT 2,3-bis-(2-methoxy-4-nitro-5-sulfophenyl)-2H-tetrazolium-5-carboxanilide (XTT) cell viability assay (ThermoFisher). Cells were plated in triplicate in a 96-well plate at 4000 cells per well (p/w) for non-differentiated and 1000 cells p/w for differentiating SKNBE2. Media-only wells were used as blanks. At various timepoints from Day 0 (D0) to Day 10 (D10), XTT solution was added, and cells were incubated for 4–6 h. Absorbance at 450 nm and 660 nm was measured on a SpectraMax iD3 microplate reader (Molecular Devices, San Jose, CA, US). Specific absorbance was calculated using the formula: specific absorbance = [Abs_450nm_(condition) – Abs_450nm_(blank)] – Abs_660nm_(condition). Experiments were repeated at least three times. Normality was assessed with the Kolmogorov–Smirnov test, followed by two-way ANOVA and Bonferroni post-hoc analysis.

### Scratch assay

To assess SKNBE2 migration, 200,000 cells were plated in silicone culture-insert chambers (ibidi, Graefelfing, Germany). The inserts were removed 24 h later, and four cell-free scratch areas were imaged at 0, 2, 4, 8 and 24 h, then measured using the MRI wound healing tool [18] on Fiji. Experiments were repeated at least three times. Normality was assessed using the Kolmogorov–Smirnov test, followed by two-way ANOVA and Bonferroni post-hoc test.

### Neurite outgrowth assay

SKNBE2 cells were plated on PDL-coated coverslips and differentiated as described above. On Day 9, cells were transfected with plasmid DNA (pcDNA3.1-mCD8-GFP) for green fluorescent protein expression using Metafectene Pro (Biontex). On Day 10, cells were washed in DPBS, fixed in 4% PFA for 20 min, and processed for ICC with DAPI (1:50,000; ThermoFisher) for nuclear staining. Images were captured with the Axio Imager Z2 microscope (Zeiss) and analysed using Zen Digital Imaging software (Zeiss). Neurite length was measured with the NeuronJ plugin in Fiji. Experiments were repeated at least three times, imaging and measuring approximately 20 cells per line per experiment. Statistical significance was assessed using a two-tailed *t*-test.

### Fly lines

All fly lines used are listed in Supplementary Table S2. The *Drosophila* RNAi Screening Center Integrative Ortholog Prediction Tool (DIOPT; https://www.flyrnai.org/diopt) [19] identified *Srrm1* as the single orthologue of *SRRM1*, distinct from *Srrm234*, which corresponds to *SRRM2, SRRM3* and *SRRM4*. *Srrm1* RNA interference (RNAi) and control lines were obtained from the Bloomington Drosophila Stock Centre (BDSC #55205, #36304) and the Vienna Drosophila Research Centre (VDRC #100751, #60100). *Drosophila* were maintained on standard fly food (sugar, cornmeal, yeast and agar). Ubiquitous, glial, pan-neuronal or motoneuronal knockdown was induced using the UAS/GAL4 system [20], with the following driver lines: actin-Gal4/Tm3SbTb (ubiquitous), BDSC #7415 repo-Gal4/TM3Sb (glial), BDSC #8765 elav-GAL4/CyO (pan-neuronal), BDSC #8751 insc-GAL4 (pan-neuronal), and BDSC #8816 D42-GAL4 (motoneuronal). Flies were crossed at 18 °C, RT, or 28 °C. *Srrm1* KD efficiency was confirmed by quantitative polymerase chain reaction (qPCR) following standard procedures after RNA extraction from larvae with ubiquitous KD. Progeny from crosses between GAL4 driver lines and BDSC #55205 or VDRC #100751 are referred to as KD1 and KD2, respectively.

### Negative geotaxis and bang sensitivity assays

200 flies per cross per assay were collected 48 h post-eclosion under CO_2_ anaesthesia. Flies were housed in groups of 10 (5 male/ 5 female) for 24 h in vials with standard food. On the assay day, flies were transferred to test vials and allowed to acclimate for 1–2 min. For geotaxis, flies were tapped to the vial base and filmed for 30 s. The time for 70% of flies to climb 8.8 cm and the fraction crossing the threshold in the first 10 s were recorded. For bang sensitivity, flies were vortexed for 10 s and filmed for the next 10 s. Three readouts were measured: the number of flies in spasm or paralysis at 2 and 5 s, and the fraction remaining at the vial base 5 s after vortex. Wilcoxon signed-rank test was used for statistical analysis.

## Results

### Molecular and clinical data

#### Individual 1

Individual 1 is a 6-year, 6-month-old girl born to non-consanguineous parents, with a healthy brother. Family history was unremarkable for neurodevelopmental or growth abnormalities, except for a maternal second cousin with severe intellectual disability. During the first two trimesters, the mother was treated with INN-adalimumab for ankylosing spondylitis. Due to severe intrauterine growth retardation and increased nuchal translucency (3.3 mm), prenatal karyotyping and chromosomal microarray analysis were performed, both yielding normal results. The girl was born small for gestational age at 37 weeks, with a weight of 1795 g (z = −2.89), a length of 43 cm (z = −2.88) and head circumference of 31 cm (z = −2.29). First noted through newborn screening, relapsing hypertyrosinemia was observed until the age of 3 years. Succinylacetone was only minimally elevated in dried blood spots, and undetectable in urine. Abdominal ultrasound repeatedly showed normal liver parenchyma without hepatomegaly or nodules. Genetic testing of *FAH*, *HPD* and *TAT* revealed normal results. Therefore, the elevated tyrosine levels were not considered clinically relevant, and no therapy was initiated. Tyrosine levels and all associated metabolites spontaneously normalised after age 3 years (Supplementary Table S3).

**Fig. 1:**
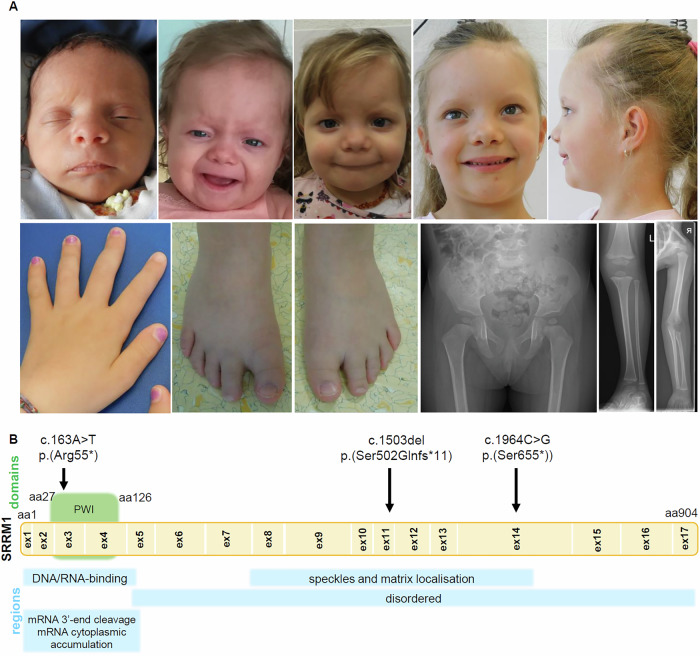
Truncating variants in *SRRM1* are associated with neurodevelopmental and growth phenotypes. **A** Clinical photographs of Individual 1 at different ages, from birth to 5.5 years. Notable facial aspects include a prominent forehead, mild hypertelorism, a bulbous nasal tip, a prominent columella, and broad, short toes. X-rays show signs of epiphyseal dysplasia, with generalised maturation delay and mild de-configuration of the epiphyses. **B** Schematic drawing of the *SRRM1* gene (NM_005839.4), its encoded domains, and functional regions [40]. Identified variants are indicated by arrows. aa amino acid, ex exon, mRNA messenger RNA, PWI proline-tryptophan-isoleucine, SRRM1 serine/arginine repetitive matrix protein 1.

Growth delay continued postnatally. At 2 years, height was 79.2 cm (z = −2.19), head circumference was 46.5 cm (z = −1.49), and weight was 10.6 kg (z = −0.76). At 5 years 11 months, height was 104 cm (z = −2.07), weight was 17.7 kg (z = −0.84), and head circumference was 49 cm (z = −1.60). At age 6.5 years height was 108 cm (z = −1.85). The girl showed subtly disproportionate stature with appearance of relatively short arms. X-rays at age 3 years revealed signs of epiphyseal dysplasia, generalised maturation delay, and mild epiphyseal de-configuration (Fig. 1A). Alkaline phosphatase was low (103–136 U/L, ref 142–335 U/L), and urinary phosphoethanolamine was slightly elevated. At age 6.5 year, the upper segment (57 cm) to lower segment (51 cm) ratio (1.12), and arm span (108 cm) to height (108 cm) ratio (1) were normal.

Also, psychomotor developmental delay was noted: Sitting age was 9 months, she did not crawl and walked only at age 2.5 years. She spoke first words at age 2 years and began speaking in sentences at 5 years. Testing with the WPPSI-IV at age 4 years did not allow an objective assessment of the cognitive developmental level. It is estimated to be in the lower normal range, with prominent impairment in expressive language, while receptive language abilities and fine motor development appeared normal.

During nursery school, she received logopaedic therapy and special needs education. Schooling has been deferred for one year and is planned to be accompanied by special needs support measures.

Dysmorphic features included hypertelorism, prominent eyes, a bulbous nasal tip, and a thin upper lip (Fig. 1A). Trio exome sequencing identified a de novo *SRRM1* variant: c.1503del, p.(Ser502Glnfs*11) (Fig. 1B, Supplementary Table S4). No other variants were found in genes associated with neurodevelopmental, metabolic, or skeletal disorders.

#### Individual 2

Individual 2 is an 11-year-old female, born at gestational week 39 with a weight of 2525 g (z = −1.95), length of 45 cm (z = −2.77) and head circumference of 32.5 cm (z = −1.62). She had developmental delay, walking and speaking at age 2 years, and has mild intellectual disability. She attended mainstream school with support until age 10 years, and then integrated into a special needs school. At age 11 years, she showed mild short stature (height: 139 cm, z = −1.96; weight: 30 kg, z = −1.94; head circumference: 52 cm, z = −1.22). Facial dysmorphism included a high forehead and bulbous nasal tip. Hypertrichosis and pectus excavatum were present. Aside from mitral and tricuspid valve insufficiency, no other anomalies were reported. Trio exome sequencing identified a de novo *SRRM1* variant, c.1964C>G, p.(Ser655*) (Fig. 1B, Supplementary Table S4).

#### Individual 3

Individual 3 is a 17-year-old male with autism, significant intellectual disability, and severe apraxia. He has a history of recurrent respiratory infections, marked right-sided lymphoedema/swelling of unknown aetiology, recurrent axillary furuncles, bronchiectasis, and atelectasis. He also experienced excessive weight gain with obesity and endocrine abnormalities, including delayed puberty, low testosterone, and mildly positive thyroperoxidase antibodies. Recurrent, unexplained episodes of non-responsiveness and possible cyanosis were reported. Facial dysmorphisms include macrocephaly, folded ear helices, synophrys, downslanting palpebral fissures, full cheeks, and retrognathia. Exome sequencing identified the variant c.163A>T, p.(Arg55*) in *SRRM1* in 28 of 70 reads (Fig. 1B, Supplementary Table S4). Parental samples were unavailable. A variant of unknown significance (p.(Met395Thr)) in *NFKB2* was also identified and may contribute to the recurrent infections and furuncles.

Supplementary Table S4 provides an overview of the clinical aspects of all three individuals. Variants p.(Ser502Glnfs*11) and p.(Ser655*), found in Individuals 1 and 2, respectively, are predicted to trigger nonsense-mediated mRNA decay. Variant p.(Arg55*) in Individual 3 is located near the N-terminus and may therefore escape nonsense-mediated mRNA decay [21, 22]. It is possible that, if an alternative start codon at amino acid position 82 is used, a truncated protein with a disrupted PWI domain and impaired function could result. *SRRM1* has a predicted loss-of-function (pLI) score of 1 and a loss-of-function observed/expected upper fraction (LOEUF) of 0.225, indicating intolerance to loss-of-function (LoF) variants [23]. Variants c.1503del and c.1964G>C are located in invariable exons that are present and coding in all 31 isoforms listed in the UCSC Genome Browser (hg38). Variant c.163A>T resides in exon 3 of the MANE Select transcript, which is present in all 31 isoforms, but is coding in only 12 of them. The remaining isoforms contain an alternative downstream start codon in exon 4. However, since the PWI domain is encoded by exons 3 and 4, these isoforms are predicted to lack at least parts of the PWI domain and may therefore be (partially) non-functional. None of the three variants is listed in the Genome Aggregation Database (gnomAD v4.1.0; https://gnomad.broadinstitute.org/) [23].

### SRRM1 expression is reduced by half after CRISPR-Cas9 editing of SKNBE2 cells

**Fig. 2:**
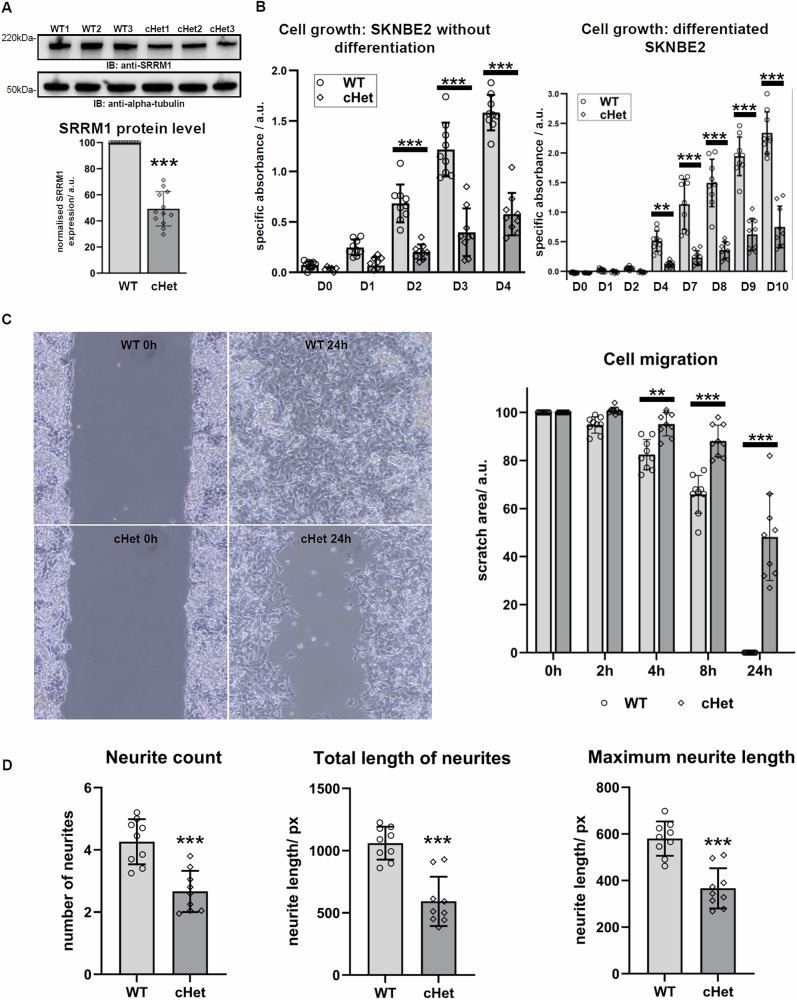
Cell proliferation, cell migration, and neurite outgrowth are impaired in SRRM1 cHet cells. **A** Representative blot showing SRRM1 expression levels in WT and SRRM1 cHet cells (top). Alpha-tubulin-normalised densitometric values for WT and cHet cells were compared using a one-sample *t*-test (hypothetical value = 100), revealing significantly lower SRRM1 expression in cHet cells than in WT cells (***p < 0.0001) (bottom). The mean SRRM1 expression in cHet cells was 49.32 (SD = 13.18); *t*(11) = −13.32, *p* < 0.0001. Each data point represents an independent WT or cHet cell line, with the experiment repeated across four runs. Densitometric measurements for each individual cell line are shown in Supplementary Fig. S1. **B** XTT assay and two-way ANOVA on non-differentiated SKNBE2 cells revealed significant effects of SRRM1 depletion (*F*(1,16) = 156.49, *p* < 0.0001), time (*F*(4,64) = 149.55, *p* < 0.0001), and their interaction (*F*(4,64) = 31.97, *p* < 0.0001). Bonferroni post hoc analysis showed reduced proliferation in cHet cells compared to WT from Day 2 (D2) onwards (***p < 0.001) (left). In differentiated SKNBE2 cells, two-way ANOVA showed significant effects of SRRM1 depletion (*F*(1,16) = 138.93, *p* < 0.0001), time (*F*(7,112) = 157.68, *p* < 0.0001), and their interaction (*F*(7,112) = 42.59, *p* < 0.0001). Bonferroni post hoc analysis revealed slower growth in SRRM1 cHet cells from Day 4 (D4) onwards (**p < 0.01 and ***p < 0.001) (right). The Kolmogorov–Smirnov test confirmed data normality (*p* > 0.10). Each data point represents an independent WT or cHet cell line, with the experiment repeated three times. Measurements for each individual cell line are shown in Supplementary Fig. S3. **C** Representative images showing the scratch area at 0 h and 24 h for WT and SRRM1 cHet cells (left). Assessment of cell migration by scratch assay and two-way ANOVA showed that SRRM1 depletion (*F*(1,16) = 70.06), time (*F*(4,64) = 463.33), and their interaction (*F*(4,64) = 42.18) each had a significant effect on migration (*p* < 0.0001 for all). Bonferroni post hoc analysis revealed significantly slower migration in SRRM1 cHet cells compared to WT from 4 h onwards (**p < 0.01, ***p < 0.001) (right). Each data point represents an independent WT or cHet cell line, with the experiment repeated three times. Measurements on each individual cell line are shown in Supplementary Fig. S4. **D** The number of primary neurites (*t*(16) = 4.873, *p* = 0.0002), total neurite length (*t*(16) = 5.889, *p* < 0.0001), and maximum neurite length (*t*(16) = 5.631, *p* < 0.0001) were significantly lower in differentiated SRRM1 cHet cells compared to WT, as determined by a two-tailed *t*-test (***p < 0.001). Each data point represents the average of at least 20 cells from an independent WT or cHet cell line, with the experiment repeated three times. Measurements for each individual cell line are shown in Supplementary Fig. S5. a-tub alpha-tubulin, a.u. arbitrary units, D day, h hour, px pixels, XTT 2,3-bis-(2-methoxy-4-nitro-5-sulfophenyl)-2H-tetrazolium-5-carboxanilide.

To assess the cellular effects of heterozygous *SRRM1* truncating variants, we used CRISPR-Cas9 to edit the SKNBE2 neuroblastoma cell line. This generated three SRRM1 compound heterozygous (cHet) lines, each carrying a truncating variant on one allele and an in-frame variant on the other (Supplementary Table S1). The in-frame deletions did not reside in the PWI domain, and deleted amino acid residues were recurrently affected by missense variants in gnomAD and predicted by MetaDome (https://stuart.radboudumc.nl/metadome/) [24] to be slightly intolerant (Pro382,Arg283), neutral (Lys384, Thr385, Arg386) or (slightly) tolerant (Arg387, Leu388, Ser389). Western blotting (WB) of cell lysates revealed an approximately 50% reduction in SRRM1 protein levels in cHet lines compared to WT (Fig. 2A). Mean relative protein expression (normalised to alpha-tubulin) in cHet cells (M = 49.32, SD = 13.18) was significantly lower than in WT, *t*(11) = −13.32, *p* < 0.0001 (one-sample *t*-test) (Fig. 2A, Supplementary Fig. S1). These lines were therefore considered a suitable model for heterozygous *SRRM1* loss, assuming a neglegible effect of the in-frame variant. We also assessed SRRM1 intracellular localisation in WT and SRRM1 cHet cells and found no significant differences (Supplementary Fig. S2A). SRRM1 remained predominantly localised to nuclear speckles (Supplementary Fig. S2B).

### Cell proliferation, migration and neurite outgrowth are impaired in SRRM1 cHet cells

To assess the effect of heterozygous SRRM1 loss on cell growth, we performed an XTT assay on non-differentiated SKNBE2 cells over time. Two-way ANOVA revealed a significant main effect of SRRM1 depletion (*F*(1,16) = 156.49, *p* < 0.0001), a significant main effect of time (*F*(4,64) = 149.55, *p* < 0.0001), and a significant interaction between SRRM1 depletion and time (*F*(4,64) = 31.97, *p* < 0.0001). Bonferroni post hoc analysis showed that cell proliferation was significantly reduced in cHet cells compared to WT cells from Day 2 (D2) onwards (*p* < 0.001) (Fig. 2B, Supplementary Fig. S3A). Similarly, in differentiating SKNBE2 cells, two-way ANOVA showed significant effects of SRRM1 depletion (*F*(1,16) = 138.93, *p* < 0.0001), time (*F*(7,112) = 157.68, *p* < 0.0001), and their interaction (*F*(7,112) = 42.59, *p* < 0.0001). Bonferroni post hoc analysis revealed slower growth in SRRM1 cHet cells from Day 4 (D4), with *p* < 0.01 at D4 and *p* < 0.001 at later timepoints (Fig. 2B, Supplementary Fig. S3B). To evaluate cell migration, we conducted a scratch assay (Fig. 2C), monitoring WT and SRRM1 cHet cells over 24 h. Two-way ANOVA showed that SRRM1 depletion, time, and their interaction each had a significant effect on cell migration (F(1,16) = 70.06, F(4,64) = 463.33, F(4,64) = 42.18; *p* < 0.0001 for all). Cell migration was significantly slower in SRRM1 cHet cells compared to WT, as shown by Bonferroni post hoc tests, with significant differences from 4 hours (4 h) onwards (*p* < 0.01 at 4 h; *p* < 0.001 at later time points) (Fig. 2C, Supplementary Fig. S4). Following 10 days of differentiation and neurite quantification, SRRM1 cHet cells displayed significantly fewer primary neurites (t(16) = 4.873, *p* = 0.0002), shorter total neurite length (t(16) = 5.889, *p* < 0.0001), and reduced maximum neurite length (t(16) = 5.631, *p* < 0.0001) compared to WT cells (Fig. 2D, Supplementary Fig. S5).

### Knockdown of *Srrm1* results in neurological dysfunction in *Drosophila*

**Fig. 3:**
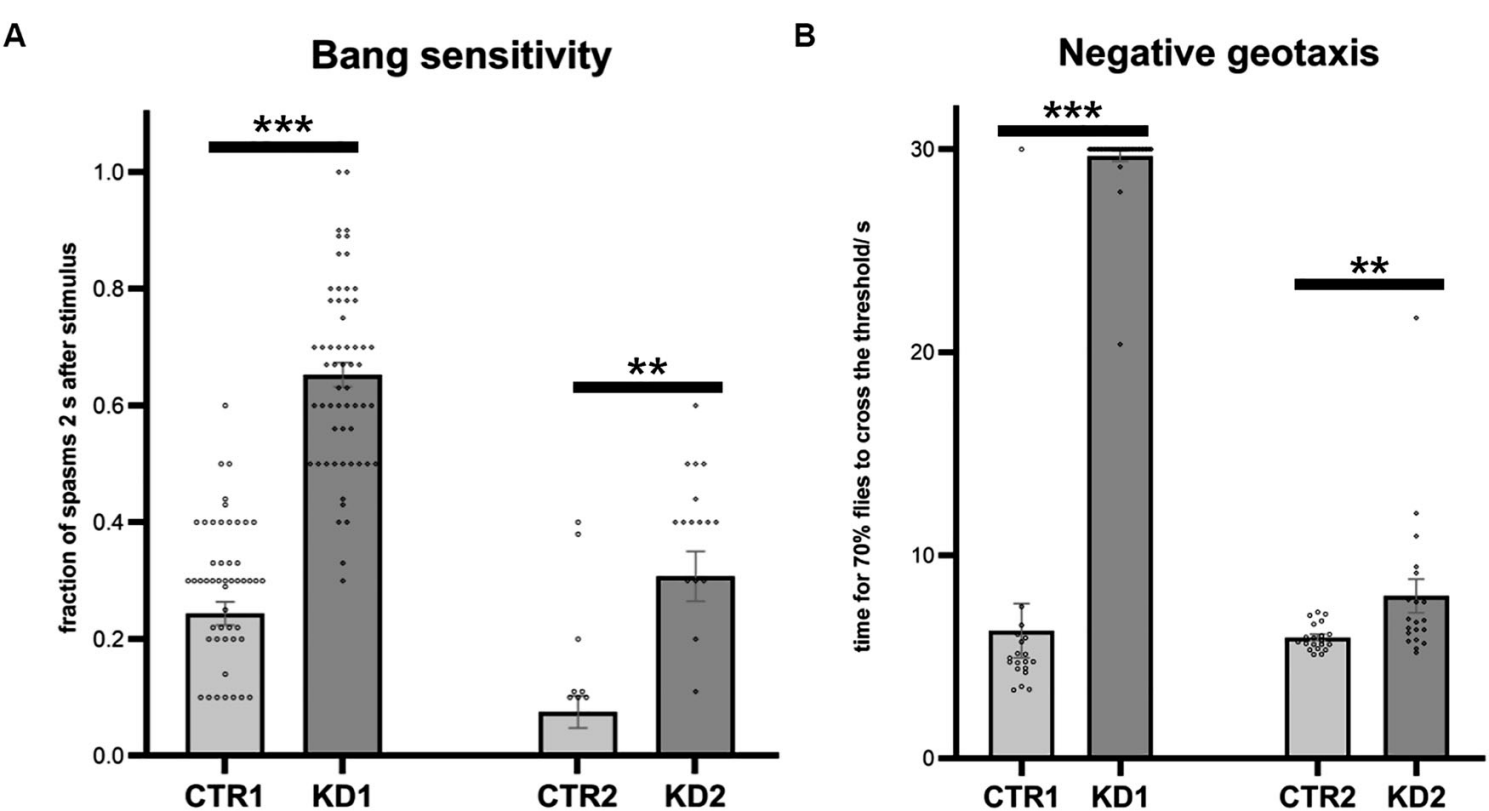
Knockdown of *Srrm1* results in neurological dysfunction in fruit flies. **A** The fraction of flies seizing 2 s after the bang stimulus was significantly higher in KD1 (*W* = −1698) and KD2 (*W* = −136) flies compared to controls, as determined by the Wilcoxon signed-rank test (KD1 *W* = −171; KD2 *W* = −148; **p < 0.01 and ***p < 0.0001). **B** The time for 70% of flies to cross the threshold in the negative geotaxis assay was significantly increased with *Srrm1* KD, as assessed by the Wilcoxon signed-rank test (**p < 0.01 and ***p < 0.001). Data are presented as mean ± standard error (SE). a.u. arbitrary units, CTR control, KD knockdown, s second, Srrm1 serine/arginine repetitive matrix protein 1.

To investigate the effects of *SRRM1* loss on the nervous system and development in vivo, we used *Drosophila melanogaster* as a model organism, in which *Srrm1* is the sole orthologue of *SRRM1*. Ubiquitous knockdown was achieved by inducing *Srrm1* RNAi using two independent lines: BDSC #55205 and VDRC #100751, hereafter referred to as KD1 and KD2, respectively. This led to a 50% reduction in *Srrm1* expression for KD1 and a 30% reduction for KD2 during the larval stage (Supplementary Fig. S6). Increased lethality was observed when *Srrm1* RNAi lines were crossed with various tissue-specific driver lines (ubiquitous, glial, pan-neuronal, motoneuronal) at 28 °C, room temperature or 18 °C (Supplementary Table S5). Ubiquitous and glial knockdown of *Srrm1* caused complete lethality at all tested temperatures. Similarly, pan-neuronal knockdown produced no viable offspring with either RNAi line at any temperature (Supplementary Table S4). In contrast, motoneuronal knockdown at 18 °C allowed the development of viable progeny. Behavioural testing of these flies using the bang sensitivity assay revealed a significant increase in the proportion of flies experiencing spasms for 2 s (KD1: *W* = −1698, *p* < 0.0001; KD2: *W* = −136, *p* =  0.0031) (Fig. 3A) and 5 s (KD1: *W* = −1570, *p* < 0.0001; KD2: *W* = −52, *p* = 0.0435) (Supplementary Fig. S7A) following stimulation, as determined by the Wilcoxon signed-rank test. The fraction of flies remaining at the bottom of the vial 5 s after the bang stimulus was also significantly higher in KD1 compared to controls (*W* = −1542, *p* < 0.0001) (Supplementary Fig. S7B). When flies with motoneuronal *Srrm1* knockdown were assessed for locomotor function using the negative geotaxis (climbing) assay, the time required for 70% of flies to cross the threshold was significantly increased compared to WT (KD1: W = −171, *p* = 0.0002; KD2: *W* = −148, *p* = 0.0061) (Fig. 3B). Furthermore, the proportion of flies crossing the threshold within 10 seconds was significantly lower in KD1 compared to controls (*W* = 190, *p* = 0.0001) (Fig. S7C). Collectively, these results suggest that knockdown of *Srrm1* leads to neurological and motor dysfunction in *Drosophila*.

## Discussion

We identified heterozygous truncating variants in *SRRM1* in three individuals with variable neurodevelopmental and growth phenotypes. Global matchmaking platforms such as GeneMatcher [15] have successfully contributed to identification and confirmation of numerous NDD genes over the past decade. However, the number of unconfirmed candidate NDD genes remains high (1753 definitive versus 1394 candidate genes in the SysNDD database (https://sysndd.dbmr.unibe.ch/) [25], as of March 2025), highlighting the challenges of establishing disease genes and genotype-phenotype correlations in ultra-rare disorders.

Although *SRRM1* cannot yet be classified as a definitive disease gene, we have gathered several lines of evidence supporting its role in NDDs. All three individuals in this study carry heterozygous truncating variants in *SRRM1*, occurring de novo in two cases. Parental testing was not available for the third case. According to gnomAD constraint scores, *SRRM1* is highly intolerant to loss-of-function variants, supporting a disease-causing role through loss of function or haploinsufficiency. In contrast, no strong intolerance to missense variants is indicated (z = 2.34) [23]. The DECIPHER database [26] reports four deletions involving *SRRM1*, but all encompass more than 20 genes, preventing specific conclusions about *SRRM1* loss. All three individuals with heterozygous truncating *SRRM1* variants exhibit variable neurodevelopmental and behavioural phenotypes, including developmental delay, intellectual disability and/or autistic features. Intrauterine growth retardation or small birth measurements, along with postnatal short stature were observed in two individuals. These two individuals also shared overlapping facial dysmorphism, including a prominent forehead and bulbous nasal tip. The third individual, who carries an N-terminal truncating variant in an exon that is coding in the major but not in all isoforms, presented a distinct phenotype of severe obesity and macrocephaly. Therefore and as de novo occurrence could not be tested, the evidence for variant p.(Arg55*) is currently the weakest. However, it may also be speculated that the phenotypic differences reflect variant-specific mechanisms of loss of function, such as haploinsufficiency due to nonsense-mediated decay versus truncation or differentially affected isoforms, depending on the variant location. Further cases and follow-up studies are, however, required. As additional features, including metabolic, endocrine or radiographic skeletal anomalies as well as obesity and macrocephaly, were each reported in only one individual, it remains currently unclear whether these findings are related to the *SRRM1* variant or to other, unrecognised causes. Recurrent infections observed in Individual 3 may be attributable to a variant of unknown significance in *NFKB2*.

SRRM1 belongs to the SRRM protein family, alongside SRRM2 [5], SRRM3 [27] and SRRM4 [28]. *SRRM1* and *SRRM2* are expressed in various tissues, while *SRRM3* and *SRRM4* are primarily expressed in the brain [29]. Both SRRM1 and SRRM2 function similarly in promoting the early stages of splicing [5]. Recently, truncating variants or microdeletions of *SRRM2* were identified in 22 individuals with a neurodevelopmental disorder [30] (MRD72, MIM#620439) (Supplementary Table S4). The phenotype included developmental delay, intellectual disability, behavioural issues, overweight, nonspecific facial dysmorphism, and variable other anomalies. Tall stature was observed in four affected individuals [30]. Due to the small number of individuals with *SRRM1* variants and the rather nonspecific phenotypes associated with either *SRRM1* or *SRRM2* variants, no definitive conclusions can currently be drawn regarding presence, extent or absence of phenotypic overlap. Other spliceosome components and mRNA processing factors have been implicated in syndromic disorders with neurodevelopmental and/or growth phenotypes, such as *SCAF4* (Fliedner-Zweier syndrome, MIM#620511) [31], *THOC6* (Beaulieu-Boycott-Innes syndrome, MIM#613680), *RNU4ATAC* (Lowry-Wood syndrome, MIM#226960; microcephalic osteodysplastic primordial dwarfism, type I, MIM#210710; Roifman syndrome, MIM#616651), *WBP4* (NEDHFDB, MIM#620852), *U2AF2* (DEVDFB, MIM#620535), *PRPF19* [32], *SART3* [33], or *GEMIN5* (NEDCAM, MIM#619333). Recently, non-coding de novo variants in *RNU4-2* (U4 small nuclear RNA), a key spliceosome component, were identified as causative for ReNU syndrome (MIM#620851), a prevalent NDD characterised by severe intellectual disability, short stature, microcephaly, and facial dysmorphism [34, 35].

To our knowledge, the role of *SRRM1* in nervous system development and function has not yet been characterised. We therefore employed two model systems to investigate the effects of *SRRM1* loss. Similar approaches have been applied to other spliceosome-associated NDDs [32, 33, 36]. Neuron-like cells differentiated from SKNBE2 neuroblastoma cells with heterozygous truncating variants – leading to a 50% reduction of SRRM1 protein – showed significant impairments in cell growth, migration and neurite development. Likewise, human neurons derived from pluripotent stem cells carrying recurrent, patient-derived missense variants in the spliceosome subunit U2AF2 showed defects in neurite outgrowth, while flies with knockdown of the orthologue U2af50 exhibited proliferation defects in the central nervous system [32]. These disturbance may not be specific for spliceosome-associated NDDs [37–39], but they may contribute to the neurodevelopmental and cognitive impairments seen in individuals with heterozygous truncating variants in *SRRM1*.

Flies with motoneuronal knockdown of the *Drosophila* orthologue *Srrm1* showed seizure susceptibility and significantly impaired gross neurological function. Although seizures were not observed in the three individuals with truncating *SRRM1* variants, they may still fall within the clinical spectrum and be linked to disruptions in cell growth, migration, and neurite development observed in the cellular model. Pan-neuronal, glial, and ubiquitous knockdown of *Srrm1* led to partial or complete lethality, underscoring its essential role in development, particularly of the nervous system. Similarly, knockdown of other NDD- and spliceosome-associated genes, such as *U2AF2/U2af50*, *PRP19/Prp19*, *SART3/Rnp4f*, or *GEMIN5/rigor mortis*, also resulted in lethality and a range of neurodevelopmental, neurological, and behavioural phenotypes in flies [32, 33, 36].

We present *SRRM1* as a candidate gene for a neurodevelopmental disorder, possibly with variable growth anomalies. Based on current genetic and functional findings, we assume haploinsufficiency and/or loss of function as the underlying cause. Identifying additional individuals with *SRRM1* variants and conducting further functional characterisation will be necessary to confirm the *SRRM1*-associated molecular and clinical spectrum, identify possible genotype-phenotype correlations, and better understand the underlying patho-mechanisms.

## Supplementary information

Supplementary data

## Data Availability

The datasets included and analysed during this study are available from the corresponding author upon request. Identified variants in *SRRM1* were submitted to ClinVar with the following accession numbers: SCV006308015, SCV006308016, SCV006308017.
