# Supplementary material for: Heterozygous loss of SRRM1 may be associated with neurodevelopmental phenotypes and anomalies in cell growth and neurite morphology
Source: Eur J Hum Genet. 2025 Oct 27;34(2):201–8. doi: 10.1038/s41431-025-01966-y (PMC12858978; doi:10.1038/s41431-025-01966-y)
Supplement: Supplementary file 1 — Supplementary data [file 41431_2025_1966_MOESM1_ESM.docx]

# SUPPLEMENTARY INFORMATION

## Supplementary Figures

**Fig. S1** **SKNBE2 SRRM1 cHet cells showed a reduction of protein expression compared to WT.** Densitometric measurement of the SRRM1 expression levels of three WT and SRRM1 cHet lines. SRRM1 cHet cells showed around 50% reduction in SRRM1 protein expression compared to WT. A representative blot and merged densitometric data are shown in Fig. 2A. a.u. = arbitrary units; cHet = compound heterozygous; SRRM1 = serine/arginine repetitive matrix protein 1; WT = wild-type


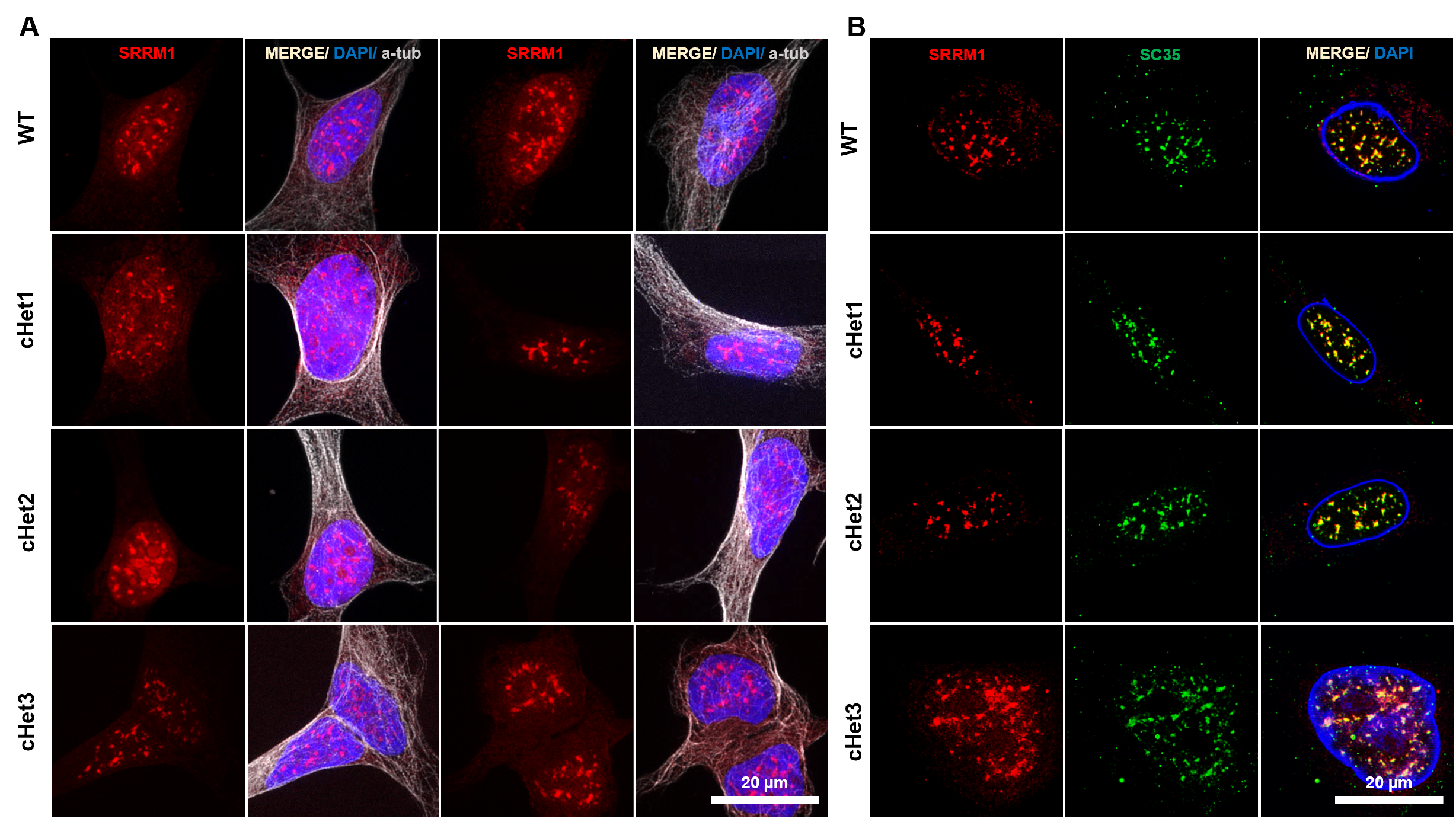


**Fig. S2** **SKNBE2 SRRM1 cHet cells showed no alteration in SRRM1 intracellular localization.** **A** SRRM1 localized to the nuclei of WT and cHet cells. **B** Nuclear SRRM1 was largely found localized to nuclear speckles, shown by the nuclear speckle marker SC35. a-tub = alpha-tubulin; cHet = compound heterozygous; DAPI = 4',6-diamidino-2-phenylindole; SC35 = serine- and arginine-rich splicing factor 2; SRRM1 = serine/arginine repetitive matrix protein 1; WT = wild-type

**Fig. S3** **Cell proliferation is impaired in SRRM1 cHet cells.** XTT assay on **A** non-differentiated and **B** differentiated SKNBE2 cells. Merged data from three different lines, each, is presented in Fig. 2B. a.u. = arbitrary units; cHet = compound heterozygous; D = day; SRRM1 = serine/arginine repetitive matrix protein 1; WT = wild-type; XTT = 2,3-bis-(2-methoxy-4-nitro-5-sulfophenyl)-2H-tetrazolium-5-carboxanilide

**Fig. S4** **Cell migration is impaired in SRRM1 cHet cells.** Assessment of cell migration via scratch assay on WT and SRRM1 cHet SKNBE2 cells. Merged data is presented in Fig. 2C. a.u. = arbitrary units; cHet = compound heterozygous; h = hour; SRRM1 = serine/arginine repetitive matrix protein 1; WT = wild-type

**Fig. S5** **Neurite outgrowth is impaired in SRRM1 cHet cells.** Number of primary neurites, the total and maximum neurite lengths measured in WT and SRRM1 cHet SKNBE2 cells. cHet = compound heterozygous; px = pixels; SRRM1 = serine/arginine repetitive matrix protein 1; WT = wild-type

**Fig. S6 *Srrm1* RNAi lines crossed with the ubiquitous actin-GAL4 driver resulted in a 50% (KD1) and 30% (KD2) reduction in *Srrm1* expression, as measured by qPCR.** KD1 refers to progeny from crosses with BDSC #55205, while KD2 refers to progeny from crosses with VDRC #100751. a.u. = arbitrary units; BDSC = Bloomington Drosophila Stock Centre; CTR = control; KD = knockdown; qPCR = quantitative polymerase chain reaction; RNAi = ribonucleic acid interference; RQ = relative quantification; Srrm1 = serine/arginine repetitive matrix protein 1; Vienna Drosophila Research Centre


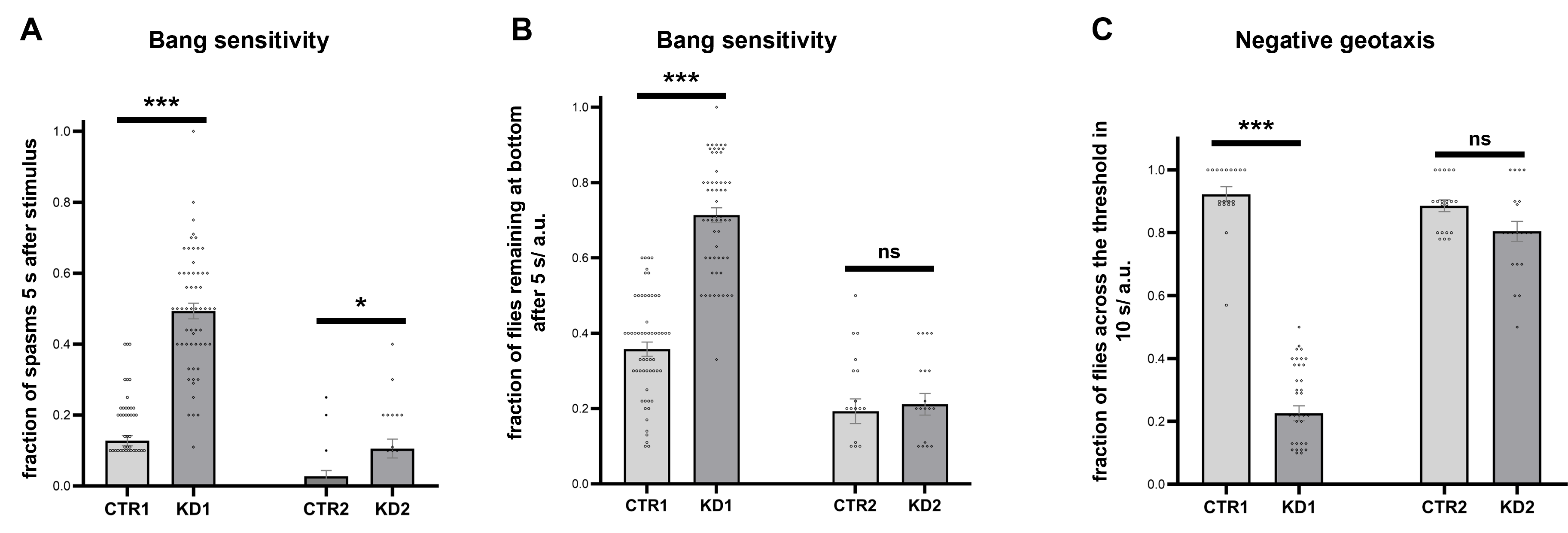


**Fig. S7 Knockdown of *Srrm1* results in neurological dysfunction in fruit flies.** **A** Fraction of flies seizing 5 s after bang stimulus was significantly higher in KD1 (*W* = -1570) and KD2 (*W* = -52) flies compared to controls in a Wilcoxon signed-rank test (***p < 0.0001 and *p < 0.05). **B** Fraction of flies remaining still at the bottom of the test vial 5 s after bang stimulus was significantly increased in KD1 flies (*W* = -1542) compared to their controls in a Wilcoxon signed-rank test (***p < 0.0001). **C** In the negative geotaxis assay, the fraction of flies crossing the threshold in 10 s was significantly lower in KD1 flies (*W* = 190) compared to their controls in a Wilcoxon signed-rank test (***p < 0.001). Data are presented as mean ± standard error (SE). a.u. = arbitrary units; CTR = control; KD = knockdown; ns = not significant; s = second; Srrm1 = serine/arginine repetitive matrix protein 1

## Supplementary Tables

**Table S1 SKNBE2 lines generated via CRISPR-Cas9 editing of *SRRM1***

| **SKNBE2 line** | **variation (bp)** | **genomic** | **protein** |
| --- | --- | --- | --- |
| WT1 | - | - | - |
| WT2 | - | - | - |
| WT3 | - | - | - |
| cHet1 | +1/-12 | NM_005839.4:c.[1155dup];[1157_1168del] | p.(Arg386Serfs*2); p.(Arg386_Ser389del) |
| cHet2 | +1/-12 | NM_005839.4:c.[1155dup];[1157_1168del] | p.(Arg386Serfs*2); p.(Arg386_Ser389del) |
| cHet3 | +1/-18 | NM_005839.4:c.[1155dup];[1144_1161del] | p.(Arg386Serfs*2); p.(Pro382_Arg387del) |

bp = base pairs; cHet = compound heterozygous; SRRM1 = serine/arginine repetitive matrix protein 1; WT = wild-type

**Table S2 List of fruit fly lines included in this study**

| **company code** | **name** | | **type** | **KD tissue** |
| --- | --- | --- | --- | --- |
| BDSC 36304 | RNAi CTR1 | | RNAi CTR | - |
| BDSC 55205 | *Srrm1* RNAi1 | | *Srrm1* RNAi | - |
| VDRC 60100 | RNAi CTR2 | | RNAi CTR | - |
| VDRC 100751 | *Srrm1* RNAi2 | | *Srrm1* RNAi | - |
| in-house | actin | | Actin-Gal4/Tm3SbTb | ubiquitous |
| BDSC #7415 | repo | | repo-GAL4/Tm3Sb | glial |
| BDSC #8765 | elav | | elav-GAL4/CyO | pan-neuronal |
| BDSC #8751 | insc | | insc-GAL4 | pan-neuronal |
| BDSC #8816 | D42 | D42-GAL4 | | motoneuronal |

BDSC = Bloomington Drosophila Stock Center; CTR = control; KD = knockdown; RNAi = ribonucleic acid interference; Srrm1 = serine/arginine repetitive matrix protein 1; VDRC = Vienna Drosophila Research Center

**Table S3 Metabolic analyses performed in Individual 1**

|  | Ref-range/U | Unit | 1 w | 2 w | 3 w | | 1 mo | | 3 mo | | 8 mo | | 11 mo | | 17 mo | |  |
| --- | --- | --- | --- | --- | --- | --- | --- | --- | --- | --- | --- | --- | --- | --- | --- | --- | --- |
| Tyrosine | 20-120 | µmol/L | 1,307 | 1,117 | 709 | | 381 | | 160 | | 56 | | 106 | | 154 | |  |
| Succinylacetone (DBS) | < 0.60 | µmol/L | 0.95 | 1.54 |  | | 0.69 | | 0.01 | | 0.00 | |  | |  | |  |
| Succinylacetone (urine) | < 0.01 | mol/molCr | < 0.01 | < 0.01 | < 0.01 | | < 0.01 | | < 0.01 | | < 0.01 | | < 0.01 | | < 0.01 | |  |
| ASAT | < 35 | U/L | 59 | 40 | 57 | | 116 | | 115 | | 46 | | 52 | | 47 | |  |
| ALAT | < 35 | U/L | 22 | 23 | 26 | | 35 | | 55 | | 19 | | 18 | | 27 | |  |
| INR | 0.7-1.2 | - | 1.15 | 1.05 | 10.5 | |  | |  | | < 1.0 | | < 1.0 | | 1.02 | |  |
| Albumin | 38-54 | g/L | 28 | 28 | 26 | | 30 | | 36 | | 37 | | 36 | | 42 | |  |
| AFP | < 9.9 | kU/L | 345,765.0 | 290,660.0 | 185,629.0 | |  | | 3,044.0 | | 23.3 | | 22.9 | | 6.9 | |  |
| 4-OH-Phenyllactate | < 0.03 | mol/molCr | 10.57 | 9.87 | 4.84 | | 0.50 | | 0.01 | | 0.00 | | 0.00 | | 0.00 | |  |
| 4-OH-Phenylpyruvate | < 0.02 | mol/molCr | 5.2 | 7.8 | 2.04 | | 0.28 | | 0.02 | | 0.00 | | 0.06 | | 0.04 | |  |
| 4-OH-Phenylacetate | < 0.06 | mol/molCr | 1.84 | 0.88 | 0.25 | | 0.08 | | 0.19 | | 0.00 | | 0.00 | | 0.00 | |  |
| d-ALA | ? | µmol/mmolCr | 12.1 | 4.4 |  | | 7.3 | |  | |  | |  | |  | |  |
|  |  |  |  |  |  | |  | |  | |  | |  | |  | |  |
|  | **Ref-range/U** | **Unit** | **23 mo** | **30 mo** | **36 mo** | | **42 mo** | | **4;2 y** | | **5;5 y** | | **5;11 y** | | **6;5 y** | |  |
| Tyrosine | 20-120 | µmol/L | 130 | 120 | 192 | | 75 | | 63 | | 78 | | 97 | | 95 | |  |
| Succinylacetone (DBS) | < 0.60 | µmol/L |  |  |  | |  | |  | |  | |  | |  | |  |
| Succinylacetone (urine) | < 0.01 | mol/molCr | < 0.01 | < 0.01 |  | |  | | < 0.01 | | < 0.01 | | < 0.01 | | < 0.01 | |  |
| ASAT | < 35 | U/L | 34 | 34 |  | |  | | 26 | | 34 | | 37 | | 28 | |  |
| ALAT | < 35 | U/L | 18 | 18 |  | |  | | 17 | | 24 | | 33 | | 23 | |  |
| INR | 0.7-1.2 | - | 1.02 | 1.06 |  | |  | |  | | < 1.00 | | 1.06 | | 1.05 | |  |
| Albumin | 38-54 | g/L | 42 | 43 |  | |  | | 37 | | 43 | | 38 | | 43 | |  |
| AFP | < 9.9 | kU/L | 7.6 |  |  | |  | | 3.4 | | 2.4 | | 2.5 | | < 2.3 | |  |
|  |  |  |  |  |  | |  | |  | |  | |  | |  | |  |
| 4-OH-Phenyllactate | < 0.03 | mol/molCr | 0.00 | 0.00 |  | |  | | 0.00 | | 0.00 | | 0.00 | | 0.00 | |  |
| 4-OH-Phenylpyruvate | < 0.02 | mol/molCr | 0.01 | 0.00 |  | |  | | 0.00 | | 0.00 | | 0.00 | | 0.00 | |  |
| 4-OH-Phenylacetate | < 0.06 | mol/molCr | 0.00 | 0.01 |  | |  | | 0.00 | | 0.01 | | 0.02 | | 0.02 | |  |
| d-ALA | ? | µmol/mmolCr |  |  |  | |  | |  | |  | |  | |  | |  |
| Creatinine | < 34 | µmol/L | always normal | |  |  | |  | |  | |  | |  | |  | |
| Calcium | 2.25-2.75 | µmol/L | always normal | |  |  |  |  |  |  |  |  |  |  |  |  |  |
| Inorganic phosphate | 1.20-2.10 | µmol/L | always normal | |  |  |  |  |  |  |  |  |  |  |  |  |  |

AFT = alpha-fetoprotein; ALAT = alanine aminotransferase; ASAT = aspartate aminotransferase; DBS = dried blood spot; INR = international normalised ratio; mo = month(s); U = unit; w = week(s); y = year(s)

**Table S4 Clinical and molecular data on three individuals with *SRRM1* truncating variants and comparison with individuals with *SRRM2* variants**

|  | **Individual 1** | **Individual 2** | **Individual 3** | ***SRRM2*, n = 22** [1] |
| --- | --- | --- | --- | --- |
| **family history** | normal | normal | unknown | unknown |
| **sex** | female | female | male | 14 male, 8 female |
| **age at last exam** | 6y6mo | 11y | 17y | 4-28y |
| **SRRM1 variant (NM_005839.4)** | c.1503del, p.(Ser502Glnfs*11) | c.1964C>G, p.(Ser655*) | c.163A>T, p.(Arg55*) | nonsense or frameshift |
| **exon** | 12 | 14 | 3 | variable exons |
| **genomic position (GRCh38) (NC_000001.11)** | g.24662679del | g.24669347C>G | g.24646718A>T |  |
| **detected by** | trio exome | trio exome | exome | (trio) exome |
| **test setting** | clinical | clinical | research (UDN IRB) |  |
| ***de novo*** | yes | yes | parents not available | 19 *de novo*, 1 inherited, 1 possible parental mosaic, 1 unknown |
| **other (*de novo*) variants** | no | no | VUS NFKB2: p.(Met395Thr) |  |
| **birth** | | | |  |
| **gestational age** | 37w | 39w | unknown |  |
| **birth weight (z)** | 1795 g (-2.89) | 2525 g (-1.95) | unknown |  |
| **birth length (z)** | 43 cm (-2.88) | 45 cm (-2.77) | unknown |  |
| **birth OFC (z)** | 31 cm (-2.29) | 32.5 cm (-1.62) | unknown |  |
| **growth at last follow-up** | | | |  |
| **height (z)** | 102.6 cm (-1.86) | 139 cm (-1.96) | 180.3 cm (0.02) | 4 tall stature |
| **weight (z)** | 17.1 kg (-0.73) | 30 kg (-1.94) | 136 kg (3.73) | 12/22 overweight/obesity, 4 hyperphagia |
| **OFC (z)** | 49 cm (-1.41) | 52 cm (-1.22) | 63 cm (4.08) | 1/22 microcephaly, 2/22 macrocephaly |
| **feeding difficulties** | no | unknown | no | 5/22 feeding difficulties |
| **development** | | | |  |
| **walked at** | 2.5y | 2y | 1y | 8/22 after 18mo, but all before 24mo |
| **first words at** | 2y | 2y | 10mo | 1-4y |
| **speech** | delayed, sentences at 5y | delayed | few sentences, apraxia (6y) | 16/19 language delay, first sentences 2-7y |
| **ID/DD** | mild (DQ 67 at 2y; IQ “lower AVG” at 3y10mo (WPPSI-IV)) | mild, special needs school | significant | 22/22 developmental delay, 16/20 mild ID (IQ 50-70), 4 no ID |
| **developmental regression** | no | no | no | not reported |
| **neurological/behavioural anomalies** | | | |  |
|  | | | |  |
| **seizures** | no | no | none, but spells of non-responsiveness & cyanosis | not reported |
| **MRI** | not done | normal | normal |  |
| **muscle tone issues** | no | no | no | 9/22 global hypotonia |
| **other neurological** | mild proximal muscular weakness & clumsiness | no | no | 5 coordination trouble/apraxia |
| **behavioural** | no | no | autism, severe apraxia | 9/22 autistic features, 6/22 ADHD |
| **sleeping** | no | no | no | unknown |
| **malformations/ organ abnormalities** | | | |  |
| **cardiac** | patent foramen ovale | mitral and tricuspid valve insufficiency | no | 1 complex congenital heart defect |
| **renal** | no | no | no | 1/22 unilateral kidney hypoplasia |
| **urogenital** | no | no | micropenis, shawl scrotum | 1/14 males micropenis, shawl scrotum |
| **gastrointestinal** | splenomegaly | no | no | not reported |
| **skeletal** | X-ray anomalies; low AP (<100U/L) | no | no | 6/22 short hands and feet, 1 scoliosis with hemivertebra |
| **eye** | no | no | no | 4 strabismus, 3 hypermetropia |
| **facial dysmorphism** | prominent forehead, hypertelorism, broad nasal bridge, bulbous nasal tip, thin upper lip, small teeth | high forehead, bulbous nasal tip | macrocephaly, full face, folded ear helices, downslanted palpebral fissures, full cheeks, prominent brows, retrognathia, synophrys | 3/22 epicanthus, 10/22 deep set eyes, 7/22 large everted ears, 9/22 bulbous nasal tip, 6/22 smooth philtrum and broad chin, 7/22 thin upper lip, 3/22 short neck, 5/22 hypotonic face |
| **other** | relapsing hypertyrosinemia (>1300µmol/L), transient liver involvement (^↑^TA, NH_3_, AFP, Lac) | hypertrichosis, pectus excavatum | recurrent respiratory infections and axillary furuncles, bronchiectasis and atelectasis, right-sided lymphoedema, hypogonadotropic hypogonadism, delayed puberty, low testosterone levels, acanthosis nigricans, mildly +TPO antibodies, leg length discrepancy |  |

ADHD = attention-deficit/hyperactivity disorder; AFP = alpha-fetoprotein; AP = alkaline phosphatase; AVG = average; DQ = developmental quotient; ID/DD = intellectual disability/ developmental delay; IQ = intelligence quotient; Lac = lactate; mo = month(s); MRI = magnetic resonance imaging; NH_3_ = ammonia; OFC = occipitofrontal circumference; SRRM1 = serine/arginine repetitive matrix protein 1; TA = transaminase; TPO = thyroid peroxidase; UDN IRB = Undiagnosed Diseases Network Institutional Review Board; VUS = variant of uncertain significance; w = week(s); y = year(s); z = z-score

**Table S5 Viability assessment of *Srrm1* RNAi lines crossed with driver lines with different tissue KD effects**

| **driver line /**  **tissue KD effect** | **crossed with RNAi line** | **cross at 28°C** | **cross at RT** | **cross at 18°C** |
| --- | --- | --- | --- | --- |
| actin /  ubiquitous | RNAi1 | lethal | lethal | lethal |
|  | RNAi2 | lethal | lethal | lethal |
| repo /  glial | RNAi1 | lethal | lethal | lethal |
|  | RNAi2 | lethal | lethal | lethal |
| elav /  pan-neuronal | RNAi1 | lethal | lethal | not tested |
|  | RNAi2 | lethal | viable | not tested |
| insc /  pan-neuronal | RNAi1 | not tested | not tested | lethal |
|  | RNAi2 | partially lethal | viable | viable |
| D42 /  motoneuronal | RNAi1 | lethal | lethal | viable |
|  | RNAi2 | viable | viable | viable |

KD = knockdown; RNAi = ribonucleic acid interference; RT = room temperature; Srrm1 = serine/arginine repetitive matrix protein 1

**Members of the Undiagnosed Diseases Network (version 31/03/2025)**

| **Full Name** | **Affiliation** |
| --- | --- |
| Aaron Quinlan | University of Utah |
| Abdul Elkadri | MCW-CW |
| Adeline Vanderver | CHOP |
| Adriana Rebelo | Miami |
| Alan H. Beggs | Harvard |
| Albert R. La Spada | UCI/CHOC |
| Alden Huang | UCLA |
| Alex Paul | WUSTL Clinical |
| Alexander Miller | Stanford |
| Ali Al-Beshri | UAB |
| Alistair Ward | University of Utah |
| Allen Bale | Yale |
| Allyn McConkie-Rosell | Duke |
| Alyssa A. Tran | BCM Clinical |
| Andrea Gropman | NIH UDP |
| Andres Vargas | UCLA |
| Andrew B. Crouse | UAB DMCC |
| Andrew Stergachis | PNW |
| Anna Hurst | UAB |
| Anna Raper | CHOP/UPenn |
| Arjun Tarakad | BCM Clinical |
| Ashley Andrews | University of Utah |
| Ashley McMinn | Vanderbilt |
| Ashok Balasubramanyam | BCM Clinical |
| Ayuko Iverson | Mount Sinai |
| Barbara N. Pusey Swerdzewski | NIH UDP |
| Beatriz Anguiano | Stanford |
| Ben Afzali | NIH UDP, NHGRI |
| Ben Solomon | NIH UDP, NHGRI |
| Beth A. Martin | Stanford |
| Bianca E. Russell | UCLA |
| Brandon M Wilk | UAB |
| Breanna Mitchell | Mayo Clinic |
| Brendan C. Lanpher | Mayo Clinic |
| Brendan H. Lee | BCM Clinical |
| Brent L. Fogel | UCLA |
| Brett Bordini | MCW-CW |
| Brett H. Graham | IU |
| Brian Corner | Vanderbilt |
| Brianna Tucker | Stanford |
| Bruce Gelb | Mount Sinai |
| Bruce Korf | UAB |
| Calum A. MacRae | Harvard |
| Camilo Toro | NIH UDP |
| Cara Skraban | CHOP |
| Carlos A. Bacino | BCM Clinical |
| Carol Oladele | Yale |
| Caroline Hendry | Yale |
| Carson A. Smith | Miami |
| Cecilia Esteves | Harvard DMCC |
| Changrui Xiao | UCI/CHOC |
| Charlotte Cunningham-Rundles | Mount Sinai |
| Chloe M. Reuter | Stanford |
| Christine M. Eng | BCM Sequencing |
| Chun-Hung Chan | [Sanford](mailto:Rachel.Li@SanfordHealth.org) |
| Colleen E. Wahl | NIH UDP |
| Corrine K. Welt | University of Utah |
| Cynthia J. Tifft | NIH UDP, NHGRI |
| Dana Kiley | WUSTL Clinical |
| Daniel J. Rader | CHOP/UPenn |
| Daniel Wegner | WUSTL Clinical |
| Danny Miller | PNW |
| Daryl A. Scott | BCM Clinical |
| Dave Viskochil | University of Utah |
| David A. Sweetser | Harvard |
| David R. Adams | NIH UDP, NHGRI |
| Deborah Barbouth | Miami |
| Deepak A. Rao | Harvard |
| Devin Oglesbee | Mayo Clinic |
| Devon Bonner | Stanford |
| Donald Basel | MCW-CW |
| Donna Novacic | NIH UDP |
| Dr. Francisco Bustos velasq | [Sanford](mailto:Rachel.Li@SanfordHealth.org) |
| Dustin Baldridge | WUSTL MOSC |
| Edward Behrens | CHOP |
| Edwin K. Silverman | Harvard |
| Elaine Seto | BCM Clinical |
| Elijah Kravets | Stanford |
| Elisabeth Rosenthal | PNW |
| Elizabeth A Worthey | UAB |
| Elizabeth A. Burke | NIH UDP, NHGRI |
| Elizabeth Blue | PNW |
| Elizabeth C. Chao | UCI/CHOC |
| Elizabeth L. Fieg | Harvard |
| Ellen F. Macnamara | NIH UDP |
| Elsa Balton | PNW |
| Emily Glanton | Harvard DMCC |
| Emily Shelkowitz | PNW |
| Emily Wang | Yale |
| Eric Allenspach | PNW |
| Eric Gayle | Mount Sinai |
| Eric Klee | Mayo Clinic |
| Eric Vilain | UCI/CHOC |
| Erin Conboy | IU |
| Erin E. Baldwin | University of Utah |
| Erin McRoy | WUSTL Clinical |
| Esteban C. Dell'Angelica | UCLA |
| Euan A. Ashley | Stanford DMCC |
| F. Sessions Cole | WUSTL DMCC |
| Filippo Pinto e Vairo | Mayo Clinic |
| Frances High | Harvard |
| Francesco Vetrini | IU |
| Francis Rossignol | NIH UDP, NHGRI |
| Fuki M. Hisama | PNW |
| Gabor Marth | University of Utah DMCC |
| Gail P. Jarvik | PNW |
| Gary D. Clark | BCM Clinical |
| George Carvalho | UCLA |
| Gerard T. Berry | Harvard |
| Ghayda Mirzaa | PNW |
| Giorgio Sirugo | CHOP/UPenn |
| Gonench Kilich | CHOP |
| Guney Bademci | Miami |
| Hector Rodrigo Mendez | Stanford |
| Heidi Wood | NIH UDP, NHGRI |
| Herman Taylor | Morehouse DMCC |
| Holly K. Tabor | Stanford |
| Hongzheng Dai | BCM Clinical |
| Hsiao-Tuan Chao | BCM Clinical |
| Hua Xu | Yale |
| Hugo J. Bellen | BCM MOSC |
| Hui Zhang | Yale |
| Ian Glass | PNW |
| Ian R. Lanza | Mayo Clinic |
| Ingrid A. Holm | Harvard |
| Isaac S. Kohane | Harvard DMCC |
| Isum Ward | [Sanford](mailto:Rachel.Li@SanfordHealth.org) |
| Ivan Chinn | BCM Clinical |
| J. Carl Pallais | Harvard |
| Jacinda B. Sampson | Stanford |
| James P. Orengo | BCM Clinical |
| James Verbsky | MCW-CW |
| Jared Sninsky | BCM Clinical |
| Jason Hom | Stanford |
| Jason Schend | [Sanford](mailto:Rachel.Li@SanfordHealth.org) |
| Jennefer N. Kohler | Stanford |
| Jennifer E. Posey | BCM Clinical |
| Jennifer Morgan | [Sanford](mailto:Rachel.Li@SanfordHealth.org) |
| Jennifer Schymick | Stanford |
| Jennifer Wambach | WUSTL Clinical |
| Jessica Douglas | Harvard |
| Jiayu Fu | NIH UDP, NHGRI |
| Jill A. Rosenfeld | BCM Clinical |
| Jimann Shin | WUSTL MOSC |
| Joan M. Stoler | Harvard |
| Joanna Jen | Mount Sinai |
| Joanna M. Gonzalez | Miami |
| John A. Phillips III | Vanderbilt |
| John Carey | University of Utah |
| John E. Gorzynski | Stanford |
| John J. Mulvihill | NIH UDP |
| Joie Davis | NIH UDP, NHGRI |
| Jonathan A. Bernstein | Stanford |
| Jordan Whitlock | UAB DMCC |
| Jose Abdenur | UCI/CHOC |
| Joseph Loscalzo | Harvard |
| Joy D. Cogan | Vanderbilt |
| Julian A. Martínez-Agosto | UCLA |
| Julie McCarrier | MCW-CW |
| Justin Alvey | University of Utah |
| Kahlen Darr | Mayo Clinic |
| Kaitlin Callaway | UAB |
| Kathleen A. Leppig | PNW |
| Kathleen Sullivan | CHOP |
| Kathy Sisco | WUSTL Clinical |
| Katrina Dipple | PNW |
| Kayla M. Treat | IU |
| Kelly Hassey | CHOP |
| Kelly Schoch | Duke |
| Kevin S. Smith | Stanford |
| Khurram Liaqat | IU |
| Kim Worley | BCM Clinical |
| Kimberly Ezell | Vanderbilt |
| Kimberly LeBlanc | Harvard DMCC |
| Kirsten Blanco | UCI/CHOC |
| Kumarie Latchman | Miami |
| Lance H. Rodan | Harvard |
| Laura Keehan | Stanford |
| Laura Pace | University of Utah |
| Laurel A. Cobban | Harvard |
| Lauren Blieden | BCM Clinical |
| Lauren C. Briere | Harvard |
| Lauren Jeffries | Yale |
| Laurens Wiel | Stanford |
| Layal F. Abi Farraj | UCLA |
| Leoyklang Petcharet | NIH UDP, NHGRI |
| LéShon Peart | Miami |
| Lili Mantcheva | IU |
| Lilianna Solnica-Krezel | WUSTL MOSC |
| Lindsay C. Burrage | BCM Clinical |
| Lindsay Mulvihill | Mayo Clinic |
| Lisa Schimmenti | Mayo Clinic |
| Lisa T. Emrick | BCM Clinical |
| Lorenzo Botto | University of Utah |
| Lorraine Potocki | BCM Clinical |
| Louise Bier | Mount Sinai |
| Lynette Rives | Vanderbilt |
| Lynne A. Wolfe | NIH UDP, NHGRI |
| Mafalda Barbosa | Mount Sinai |
| Maija-Rikka Steenari | UCI/CHOC |
| Manish J. Butte | UCLA |
| Manisha Balwani | Mount Sinai |
| Margaret Delgado | NIH UDP, NHGRI |
| María José Ortuño Romero | Yale |
| Maria T. Acosta | NIH UDP |
| Marie Morimoto | NIH UDP, NHGRI |
| Mariko Nakano-Okuno | UAB DMCC |
| Mariya Shadrina | Mount Sinai |
| Mark Gerstein | Yale |
| Mark Wener | PNW |
| Marla Sabaii | NIH UDP, NHGRI |
| Martha Horike-Pyne | PNW |
| Martin G. Martin | UCLA |
| Martin Rodriguez | UAB |
| Matt Velinder | University of Utah |
| Matthew Coggins | Harvard |
| Matthew Might | UAB DMCC |
| Matthew T. Wheeler | Stanford |
| MayChristine V. Malicdan | NIH UDP, NHGRI |
| Megan Bell | [Sanford](mailto:Rachel.Li@SanfordHealth.org) |
| Meghan C. Halley | Stanford |
| Melissa Walker | Harvard |
| Mia Levanto | Stanford |
| Michael Bamshad | PNW |
| Michael F. Wangler | BCM MOSC |
| Michael Muriello | MCW-CW |
| Michael Zimmermann | MCW-CW |
| Michele Spencer-Manzon | Yale |
| Miranda Leitheiser | [Sanford](mailto:Rachel.Li@SanfordHealth.org) |
| Mohamad Mikati | Duke |
| Mohamad Saifeddine | [Sanford](mailto:Rachel.Li@SanfordHealth.org) |
| Monika Weisz Hubshman | BCM Clinical |
| Monkol Lek | Yale |
| Monte Westerfield | UO MOSC |
| Mustafa Tekin | Miami |
| Nada Derar | Yale |
| Naghmeh Dorrani | UCLA |
| Neil H. Parker | UCLA |
| Neil Hanchard | NIH UDP, NHGRI |
| Nicholas Borja | Miami |
| Nicola Longo | University of Utah |
| Nicole M. Walley | Duke |
| Nitsuh K. Dargie | PNW |
| Odelya Kaufman | Yale |
| Oguz Kanca | BCM MOSC |
| Orpa Jean-Marie | NIH UDP, NHGRI |
| Page C. Goddard | Stanford |
| Paolo Moretti | University of Utah |
| Patricia A. Ward | BCM Sequencing |
| Patricia Dickson | WUSTL Clinical |
| Paul Berger | [Sanford](mailto:Rachel.Li@SanfordHealth.org) |
| Paul G. Fisher | Stanford |
| Pengfei Liu | BCM Sequencing |
| Peter Byers | PNW |
| Pinar Bayrak-Toydemir | University of Utah/ARUP |
| Precilla D'Souza | NIH UDP |
| Queenie Tan | Mayo Clinic |
| Rachel A. Ungar | Stanford |
| Rachel Evard | Mount Sinai |
| Rachel Li | [Sanford](mailto:Rachel.Li@SanfordHealth.org) |
| Rachel Mahoney | Harvard DMCC |
| Rakale C. Quarells | Morehouse DMCC |
| Ramakrishnan Rajagopalan | CHOP |
| Raquel L. Alvarez | Stanford |
| Rebecca C. Spillmann | Duke |
| Rebecca Ganetzky | CHOP |
| Rebecca Overbury | University of Utah |
| Rebekah Barrick | UCI/CHOC |
| Richard A. Lewis | BCM Clinical |
| Richard Chang | UCI/CHOC |
| Richard L. Maas | Harvard |
| Rizwan Hamid | Vanderbilt |
| Rong Mao | University of Utah/ARUP |
| Ronit Marom | BCM Clinical |
| Rosario I. Corona | UCLA |
| Runjun Kumar | PNW |
| Russell Butterfield | University of Utah |
| Sanaz Attaripour | UCI/CHOC |
| Sandesh Nagamani | BCM Clinical |
| Sara Emami | Stanford |
| Saskia Shuman | Mount Sinai |
| Seema R. Lalani | BCM Clinical |
| Serena Neumann | Vanderbilt |
| Seth Perlman | PNW |
| Shamika Ketkar | BCM Clinical |
| Shamil R. Sunyaev | Harvard DMCC |
| Shilpa N. Kobren | Harvard DMCC |
| Shinya Yamamoto | BCM MOSC |
| Shrikant Mane | Yale |
| Shruti Marwaha | Stanford |
| Sirisak Chanprasert | PNW |
| Stanley F. Nelson | UCLA |
| Stephan Zuchner | Miami |
| Stephanie Bivona | Miami |
| Stephanie M. Ware | IU |
| Stephen B Montgomery | Stanford |
| Stephen C. Pak | WUSTL MOSC |
| Steven Boyden | University of Utah |
| Suha Bachir | Stanford |
| Surendra Dasari | Mayo Clinic |
| Susan Korrick | Harvard |
| Susan Shin | Mount Sinai |
| Suzanne Sandmeyer | UCI/CHOC |
| Tahseen Mozaffar | UCI/CHOC |
| Tammi Skelton | UAB |
| Tanner D Jensen | Stanford |
| Tarun KK Mamidi | UAB |
| Taylor Beagle | [Sanford](mailto:Rachel.Li@SanfordHealth.org) |
| Taylor Maurer | Stanford |
| Teodoro Jerves Serrano | Yale |
| Terra R. Coakley | Stanford |
| Thomas Cassini | Vanderbilt |
| Thomas J. Nicholas | University of Utah |
| Timothy Schedl | WUSTL MOSC |
| Tiphanie P. Vogel | BCM Clinical |
| Vaidehi Jobanputra | Columbia |
| Valerie V. Maduro | NIH UDP |
| Vandana Shashi | Duke |
| Vasilis Vasiliou | Yale |
| Virginia Sybert | PNW |
| Vishnu Cuddapah | CHOP |
| Wendy Introne | NIH UDP, NHGRI |
| Wendy Raskind | PNW |
| Willa Thorson | Miami |
| William A. Gahl | NIH UDP, NHGRI |
| William E. Byrd | UAB DMCC |
| William J. Craigen | BCM Clinical |
| Winston Halstead | Yale |
| Yan Huang | NIH UDP, NHGRI |
| Yigit Karasozen | UCLA |
| Yong-Hui Jiang | Yale |

**References**

1. Cuinat S, Nizon M, Isidor B, Stegmann A, van Jaarsveld RH, van Gassen KL, et al. Loss-of-function variants in SRRM2 cause a neurodevelopmental disorder. Genetics in Medicine. 2022 Aug;24(8):1774–80.
